# A rare cause of cervicomediastinal cellulitis: Oesophageal perforation case report

**DOI:** 10.1016/j.amsu.2021.102195

**Published:** 2021-03-08

**Authors:** Boutaina Merzouqi, Khadija El Bouhmadi, Najib Zouhair, Youssef Oukessou, Sami Rouadi, Redallah Larbi Abada, Mohamed Roubal, Mohamed Mahtar

**Affiliations:** Department of Otolaryngology, Head and Neck Surgery, 20 August 1953 Hospital, Casablanca, Morocco

**Keywords:** Cervical esophagus, Perforation, Dysphagia, Surgery, Cellulitis

## Abstract

Esophageal perforation following an impacted foreign body (FB) is a rare and potentially life-threatening condition. Early clinical suspicion and imaging are important for a targeted management to achieve a good outcome. Endoscopic extraction of esophageal FB is a good and safe treatment alternative while the surgical procedure remains a necessary option for many patients.

We present the case of a 50 years old woman, with no relevant medical history, who accidently ingested a chicken bone during a meal causing mild dysphagia. The patient consulted immediately but was reassured after normal clinical examination. We received the patient 9 days later with severe dysphagia and cervicomediastinal cellulitis. The cervical CT scan showed the significant collection and the FB impacted in the cervical esophagus wall. A first endoscopic exploration drained the pus and allowed the placement of a nasogastric tube. However, the removal of the FB required an open cervical surgery with the evacuation of the collection and the suture of the esophageal perforation followed by the placement of a drainage tube. The patient medical state improved rapidly and no further incidents were noted.

The diagnosis of esophageal FB should be meticulous in order to avoid such life-threatening complications.

## Highlights

• Esophageal perforation secondary to an impacted foreign body is a rare and potentially life-threatening condition.
• Early clinical suspicion and imaging are important for a targeted management.
• Endoscopic extraction of esophageal foreign body is a good and safe treatment alternative that should be considered first.
• Surgical procedure, even more invasive, remains a necessary option for many patients.

## 1 Introduction

Management of ingested foreign bodies (FB) is a frequent clinical routine. Lingual tonsils, the base of tongue and cervical esophagus are the most common sites of FB impaction [1]. The most frequent ingested FBs in the upper digestive tract are chicken and fish bones, and they are the most commonly associated with pharyngo-esophageal perforation, cervical abscess and potentially life-threatening complications [2].

These FB are usually treated by a nonoperative approach via endoscopic extraction, however a surgical procedure can be required in some cases. The good outcome and prevention of complication is mainly related to the earliness of the diagnosis and treatment.

We present a case treated in our Otorhinolaryngology department of the August 20, 1953 Hospital, that supplements and supports the literature and expose a rare complication of esophageal foreign bodies.

## 2 Presentation of the case

A 50-year-old woman, with no relevant past medical history, accidently ingested a chicken bone during a meal causing her pharyngeal discomfort and mild dysphagia. The patient consulted immediately but, seemingly, after normal neck examination and normal cervical ultrasound, she was reassured and discharged with local medical treatment.

**Fig. 1:**
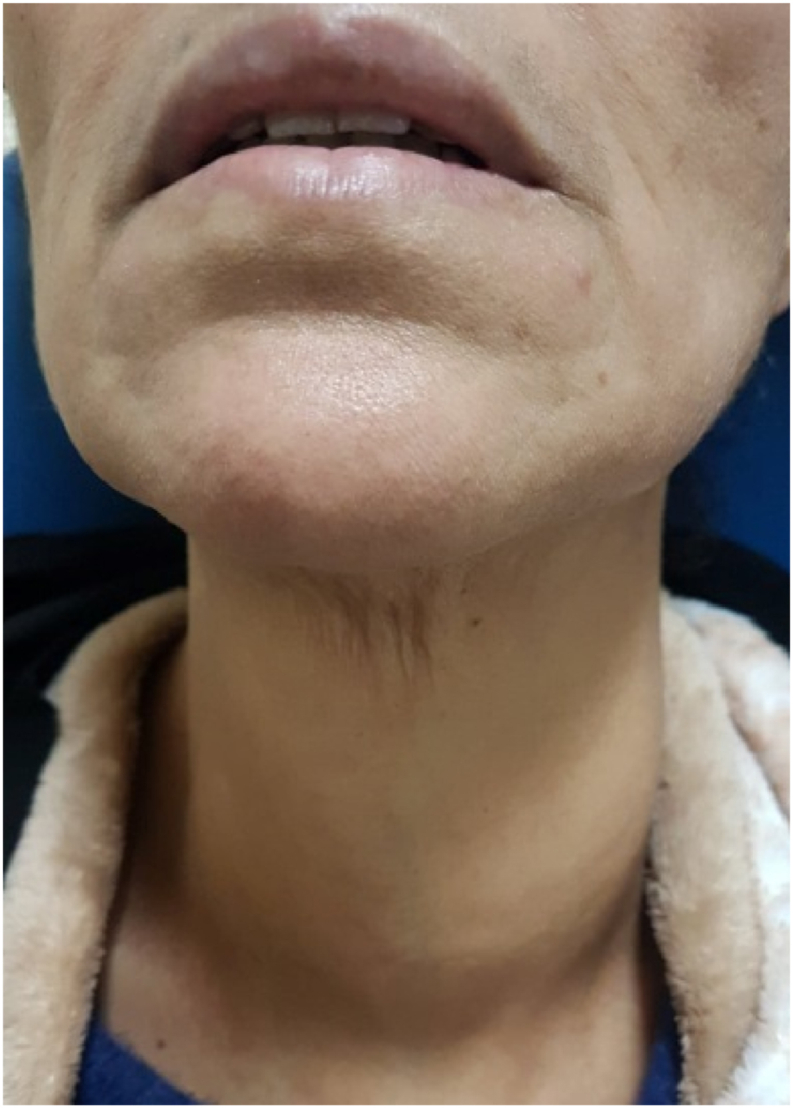
Left lateral cervical painful swelling.

We received the patient 9 days later, sent in an ambulance from her local hospital, physically altered, with severe dysphagia, substernal pain and mild fever at 38 °C. She lost an estimated weight of 5 Kgs. We discovered the manipulation of the foreign body by the patient following the persistent pharyngeal discomfort, trying vainly to extract it through her mouth. The clinical examination found no abnormalities in the oral cavity except abundant saliva. Neck examination revealed left lateral cervical painful and tender swelling, without inflammatory signs or crepitation, evolving over the past 3 days (Fig. 1).

**Fig. 2:**
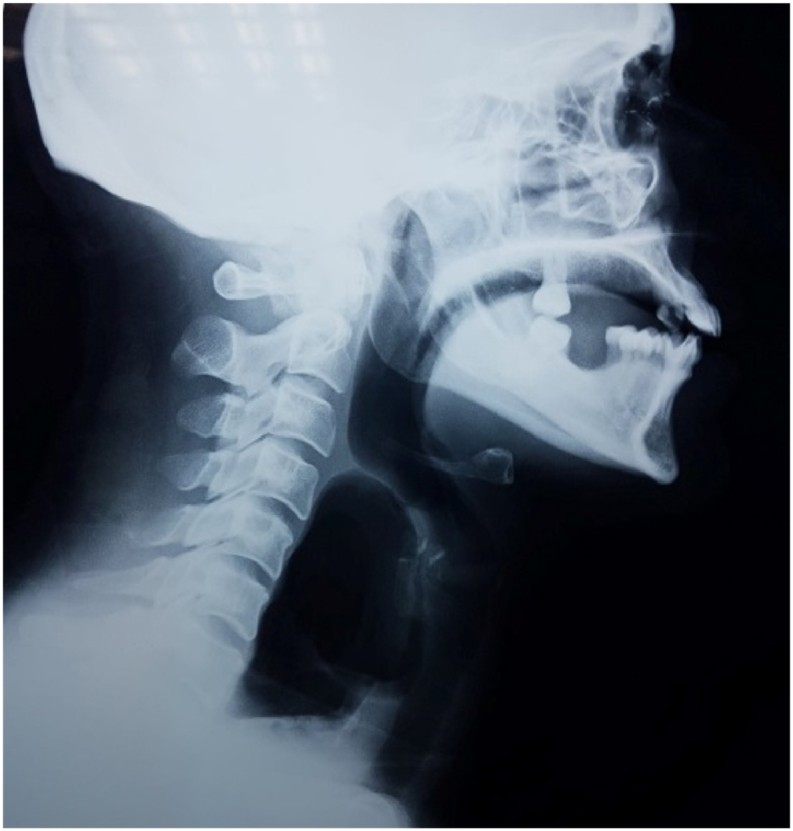
Air density cavity in the retropharyngeal and *retro*-esophageal spaces.

Cervical X ray showed a posterior air density cavity in the retropharyngeal space extending to the superior mediastinum repressing the laryngotracheal tube anteriorly (Fig. 2).

**Fig. 3:**
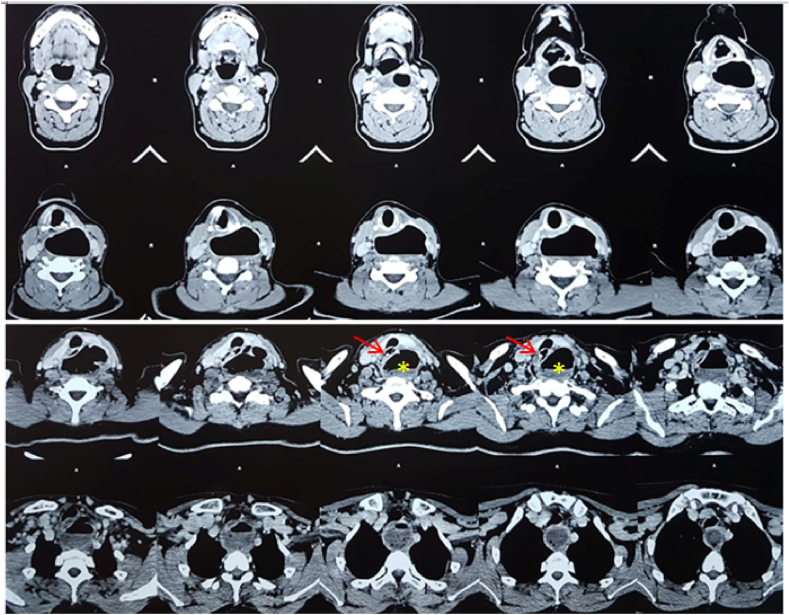
Axial CT scan sections showing the posterior collection with hydroaeric level (yellow *), its extension in the superior mediastinum (orange frame) and the foreign body appearing in calcific density enclosed in the posterior esophageal wall (red arrow).

Cervical CT scan showed a voluminous cervical posterior collection in the left retroesophageal space, measuring 60*42*82 mm, with hydroaeric level and peripheral enhancement after contrast agent injection. The collection was communicating with the posterior wall of the esophagus through a canal at the level of C5–C6. Anteriorly, the collection was repressing the larynx, trachea and left thyroid lobe. The vascular axis and the sternocleidomastoid muscle were declined laterally and the esophagus medially to the right side. Inferiorly, the collection extended to the upper mediastinum. The foreign body appeared as a bone fragment with hyperdense and calcific density (>1000UH), measuring 24*5.4 mm, in the upper part of the posterior esophagus wall, partially enclosed in its mucosa (Fig. 3).

The patient was admitted immediately. She received IV antibiotics (ceftriaxon and metronidazole) and serum glucose perfusion regarding the dysphagia. Then, she underwent an endoscopic exploration under sedation by an attending physician. The procedure found a massive collection with pus discharge that was drained by suction. The cervical mass collapsed significantly. Neither the foreign body nor the esophageal perforation was seen given the extensive inflammation of the mucosa and the abundant purulent collection. A nasogastric tube was placed under visual control for feeding. The patient was then transferred to intensive care unit, under IV empiric broad-spectrum intravenous antibiotics (ceftriaxon 3 g x 2/d + metronidazole 500 × 3/d) and analgesics.

**Fig. 4:**
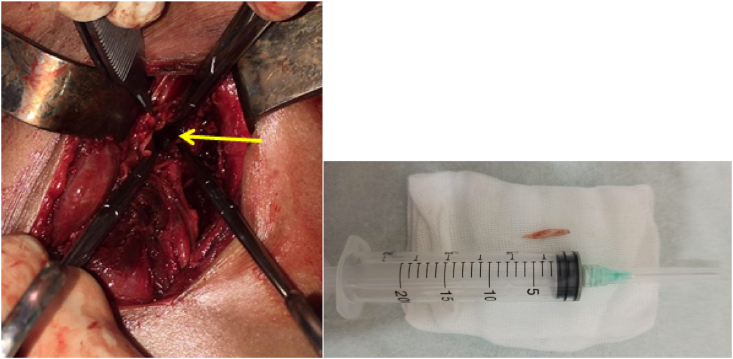
a. Esophageal perforation. (Yellow arrow) b. The foreign body, a sharp chicken bone. (For interpretation of the references to colour in this figure legend, the reader is referred to the Web version of this article.)

After a second cervical and thoracic CT scan showing the persistence of the collection, the patient was reoperated 3 days later by the same surgical team, under general anesthesia. A large left lateral cervical incision was performed and the procedure consisted on, once the collection exposed, to externally drain the pus from the cervical spaces, with retropharyngeal space dissection in order to evacuate also the upper mediastinal part. Pus samples were taken for bacteriological analysis. Then, the foreign body impacted in the upper esophageal wall was extracted by a forceps, exposing a 25 mm perforation at the site of the impaction (Fig. 4a). The edges of the perforation were derided. The entire surgical field was irrigated and washed out by Betadine solution before closing the perforation by absorbable monofilament suture in 2 layers and placing of a drainage tube in the initial site of the collection. The nasogastric tube was kept in place. And the foreign body was a sharp chicken bone of 15mm (Fig. 4b).

The patient's medical state improved daily. The cervical swelling disappeared. All the laboratory exams were normalized 3 days later. The drainage tube was removed at day 5 postoperatively. Streptococcus β haemolytic was isolated from the cultured pus, allowing for switching to amoxicillin and clavulanic acid 3g/d from day 4 and maintained for 7 more days with a daily temperature monitoring. The patient kept apyretic throughout the hospitalization duration and was discharged after 8 days. The nasogastric tube was removed two weeks later. No further incident was noted and oral alimentation was taken back progressively.

This case has been reported in line with the SCARE 2020 criteria [3].

## 3 Discussion

The upper digestive tract perforation (pharynx and cervical esophagus) is the most frequent complication related to FB's ingestion. Among the esophageal perforation etiologies, FB represent the second most common etiology after iatrogenic manipulation (esophagoscopy, esophageal dilatation, para-esophageal surgery, external trauma) [4].

Initially, clinical signs of esophageal injuries are unremarkable and usually become evident after 24 hours. Symptoms and physical examination findings vary according to the cause, localization and time elapsed between the occurrence and the diagnosis of the perforation. The most frequent symptoms are pain, fever, dysphagia, dyspnea and subcutaneous emphysema [5].

Early diagnosis is based on clinical findings, suggestive history of sharp bodies'ingestion and radiological confirmation. Direct X-ray provides important clues for the diagnosis of esophageal perforation in 70–90% of the cases [6,7]. Esophagography is necessary in all cases of esophageal perforation especially those involving the middle or the lower part of esophagus so as to confirm the diagnosis, to localize the perforation and its communication into cervical and mediastinal spaces and to provide valuable indications for treatment [5,8].

CT scan with contrast agent is another option for diagnosis. It identifies the lesion level and, if present, the foreign body, also it reveals complications related to the esophageal perforation as pneumothorax, pneumomediastinum, subcutaneous emphysema, abscess cavities. CT may even detect a very small extravasation of contrast agent into surrounding spaces (neck, mediastinal, and pleural space) showing the exact extent of peri-esophageal infection [7,9].

The gold standard of diagnosis is direct endoscopy under general anesthesia. It allows the detection of both the perforation level and the causal agent. Esophagoscopy contributes to the decision of the therapeutic method as well [5,10].

The goal of treatment is to restore the esophageal lumen and to prevent sepsis by controlling extraluminal contamination. Meanwhile, proper hemodynamic monitoring, nutritional support and systemic antibiotic therapy are mandatory [5].

Successful treatment of esophageal perforation depends on several factors including the localization and size of the rupture, the degree of contamination, and the general status of the patient, with the time period elapsed between rupture and diagnosis being the most important factor affecting the outcome of esophageal perforation [11].

Endoscopic treatment is increasingly used for the treatment of early diagnosed cases of small perforation without sign of sepsis, while perforations with large cervical, mediastinal or pleural contamination frequently require surgical treatment [5]. In these cases, surgical suture and drainage of the different affected spaces must be performed [12]. The choice of surgical approach for mediastinal drainage is dependent on abscess localization. In case of posterior and superior mediastinitis, drainage from cervical incision with retropharyngeal space dissection is adopted. This was the procedure we opted for our patient to drain both cervical and mediastinal collection through cervical route. Finally, the prognosis in case of cervical esophagus perforation is relatively good with mortality inferior to 10% [13].

## 4 Conclusion

Esophageal perforation is a rare and potentially life-threatening condition. Early clinical suspicion and imaging is important for a targeted management to achieve a good outcome. CT-scan enables an accurate and timely diagnosis and contributes to treatment indications. Extraction of esophageal FB with a rigid endoscope is a good and safe treatment alternative when performed by a trained operator. Surgical treatment remains an important option for many patients, but a nonoperative approach, through endoscopic route, should be considered when the clinical situation allows for a less invasive approach.

Also, clinicians should insist on the importance of lifestyle advices to prevent occurrence and recurrence of chicken bone ingestion starting with slow eating, efficient chewing and immediate emergency consultation in case of accidental ingestion.

## Declaration of conflicts of interest

None.

## Source of funding

None.

## Ethical approval

Obtained.

The patient gave informed consent for publication.

## Research registration unique identifying number (UIN)

Not needed.

## Author contribution

Merzouqi Boutaina: Corresponding author, writing the paper.

El Bouhmadi Khadija: Writing the paper.

Zouhair Najib: Writing the paper.

Oukessou Youssef: Study concept.

Rouadi Sami: Study concept.

Abada Redallah Larbi: Study concept.

Roubal Mohamed: Correction of the paper.

Mahtar Mohamed: Correction of the paper.

## Guarantor

Merzouqi Boutaina.

## Provenance and peer review

Not commissioned, externally peer-reviewed.

## References

[1] Chee L.W., Sethi D.S. (1999). Diagnostic and therapeutic approach to migrating foreign bodies. Ann. Otol. Rhinol. Laryngol..

[2] Selivanov V., Sheldon G.F., Cello J.P., Crass R.A. (1984). Management of foreign body ingestion. Ann. Surg..

[3] Agha R.A., Franchi T., Sohrabi C., Mathew G., for the SCARE Group (2020). The SCARE 2020 guideline: updating consensus surgical CAse REport (SCARE) guidelines. Int. J. Surg..

[4] Altorjay A., Kiss J., Voros A., Bohak A. (1997). Nonoperative management of esophageal perforations. Is it justified?. Ann. Surg..

[5] Eroğlu A., Aydın Y., Yılmaz Ö. (2018). Minimally invasive management of esophageal perforation. Turkish Journal of Thoracic and Cardiovascular Surgery.

[6] Eroglu A., Aydin Y., Yilmaz O. (2018). Thoracic perforations-surgical techniques. Ann. Transl. Med..

[7] Faggian A., Berritto D., Iacobellis F., Reginelli A., Cappabianca S., Grassi R. (2016). Imaging patients with alimentary tract perforation: literature review. Semin. Ultrasound CT MR.

[8] De Lutio di Castelguidone E., Pinto A., Merola S., Stavolo C., Romano L. (2005). Role of Spiral and Multislice Computed Tomography in the evaluation of traumatic and spontaneous oesophageal perforation. Our experience. Radiol. Med..

[9] Exarhos D.N., Malagari K., Tsatalou E.G., Benakis S.V., Peppas C., Kotanidou A., Chondros D., Roussos C. (2005). Acute mediastinitis: spectrum of computed tomography findings. Eur. Radiol..

[10] Eroglu A., Turkyilmaz A., Aydin Y., Yekeler E., Karaoglanoglu N. (2009). Current management of esophageal perforation: 20 years experience. Dis. Esophagus.

[11] Ben-David K., Behrns K., Hochwald S., Rossidis G., Caban A., Crippen C. (2014). Esophageal perforation management using a multidisciplinary minimally invasive treatment algorithm. J. Am. Coll. Surg..

[12] Righini C.A., Motto E., Ferretti G., Boubagra K., Soriano E., Reyt E. (2007). Diffuse cervical cellulites and descending necrotizing mediastinitis. Ann Otolaryngol Chir Cervicofac.

[13] Michel L., Grillo H.C., Malt R.A. (1981). Operative and nonoperative management of esophageal perforations. Ann. Surg..

